# Impact of adherence to the Mediterranean diet on stroke risk

**DOI:** 10.1007/s11357-024-01491-8

**Published:** 2025-01-07

**Authors:** Zoltan Ungvari, Mónika Fekete, Péter Varga, János Tibor Fekete, Annamaria Buda, Ágnes Szappanos, Andrea Lehoczki, Noémi Mózes, Giuseppe Grosso, Otilia Menyhart, Gyöngyi Munkácsy, Stefano Tarantini, Andriy Yabluchanskiy, Anna Ungvari, Balázs Győrffy

**Affiliations:** 1 Vascular Cognitive Impairment, Neurodegeneration and Healthy Brain Aging Program, Department of Neurosurgery, University of Oklahoma Health Sciences Center, Oklahoma City, OK USA; 2 Stephenson Cancer Center, University of Oklahoma, Oklahoma City, OK USA; 3 Oklahoma Center for Geroscience and Healthy Brain Aging, University of Oklahoma Health Sciences Center, Oklahoma City, OK USA; 4 Department of Health Promotion Sciences, College of Public Health, University of Oklahoma Health Sciences Center, Oklahoma City, OK USA; 5 International Training Program in Geroscience, Doctoral College/Institute of Preventive Medicine and Public Health, Semmelweis University, Budapest, Hungary; 6 Institute of Preventive Medicine and Public Health, Semmelweis University, Budapest, Hungary; 7 Dept. of Bioinformatics, Semmelweis University, 1094 Budapest, Hungary; 8 Cancer Biomarker Research Group, Institute of Molecular Life Sciences, HUN-REN Research Centre for Natural Sciences, 1117 Budapest, Hungary; 9 Doctoral College, Health Sciences Program, Semmelweis University, Budapest, Hungary; 10 Department of Vascular and Endovascular Surgery, Heart and Vascular Center, Semmelweis University, Budapest, Hungary; 11 Department of Rheumatology and Clinical Immunology, Semmelweis University, Budapest, Hungary; 12 Department of Biomedical and Biotechnological Sciences, University of Catania, Catania, Italy; 13 Center for Human Nutrition and Mediterranean Foods (NUTREA), University of Catania, Catania, Italy; 14 Department of Biophysics, Medical School, University of Pecs, 7624 Pecs, Hungary

**Keywords:** Meta-analysis, Case–control, Stroke prevention, Dietary intervention, Diet

## Abstract

Stroke is a leading cause of morbidity and mortality worldwide, and dietary patterns have emerged as a significant modifiable factor in stroke prevention. The Mediterranean diet, characterized by high intake of fruits, vegetables, whole grains, nuts, olive oil, and fish, has been widely recognized for its cardiovascular benefits. However, its specific impact on stroke risk requires further elucidation. We conducted a comprehensive meta-analysis of 30 studies, including both cohort and case–control designs, to evaluate the relationship between adherence to the Mediterranean diet and the risk of stroke. A systematic search was performed across multiple databases, and a random-effects model was used to estimate pooled hazard ratios (HRs) with 95% confidence intervals (CIs). Heterogeneity was assessed using the *I*^2^ statistic, and publication bias was examined through funnel plots and Egger’s regression test. Additionally, trial sequential analysis was conducted to determine the adequacy of the sample size. The meta-analysis revealed a significant reduction in stroke risk among individuals adhering to the Mediterranean diet, with a pooled HR of 0.88 (95% CI: 0.84–0.91). Notably, a significant heterogeneity was detected (*I*^2^ = 34%). The Z-score plot from trial sequential analysis confirmed that the sample sizes were sufficient to draw definitive conclusions. However, a potential publication bias was identified. The case–control studies confirmed a highly significant effect (HR = 0.54, 95% CI: 0.4–0.73). The funnel plots in both settings hinted at the presence of a potential publication bias, supported by a significant Egger’s test. Our findings provide robust evidence supporting the protective effect of the Mediterranean diet against stroke. Despite the presence of some heterogeneity and potential publication bias, the cumulative evidence suggests that promoting the Mediterranean diet could serve as an effective public health strategy for stroke prevention. Further research is recommended to explore the underlying mechanisms and to assess the diet’s impact across diverse populations.

## Introduction

Stroke is a leading cause of morbidity and mortality globally, with significant public health implications [1–3]. As an age-related disease, the incidence and prevalence of stroke increase sharply with advancing age, making it a growing concern in aging populations across the globe [1–3]. The aging demographics in many parts of the world are expected to significantly exacerbate the stroke burden, underscoring the urgent need for effective prevention strategies. In the USA, stroke is the fifth leading cause of death, accounting for approximately 1 in every 19 deaths [1]. Each year, around 795,000 Americans suffer a stroke, with nearly 610,000 of these being first or new strokes [4–6]. In Europe, the situation is equally concerning, with stroke being the second leading cause of death and a leading cause of adult disability [7]. The European Union alone witnesses more than 1 million strokes annually, a figure that is expected to rise as the population ages [7]. The prevalence of stroke underscores its significant impact, with nearly 7 million adults in the USA living with the aftermath of a stroke, many of whom endure chronic disabilities that severely diminish their quality of life. Similarly, in Europe, millions of individuals are affected by stroke, resulting in profound personal and societal costs [7]. The economic impact of stroke is staggering, with the total cost in the EU estimated at around €45 billion annually, including healthcare expenses, informal care, and productivity losses. These statistics highlight the critical need for effective prevention strategies that can mitigate the growing burden of stroke.

Traditionally, the primary risk factors for stroke have included hypertension, smoking, and diabetes [3, 6]. However, recent research has increasingly focused on the role of dietary patterns in stroke prevention [3], with particular emphasis on the Mediterranean diet [8–15]. This diet, characterized by a high intake of fruits, vegetables, whole grains, legumes, nuts, olive oil, and fish [16], has gained widespread recognition for its cardiovascular benefits [17–20]. Unlike other dietary approaches, the Mediterranean diet emphasizes monounsaturated fats, moderate alcohol consumption, and minimal intake of red meat. These dietary components are associated with a range of health benefits [21–29], particularly in reducing the risk of cardiovascular diseases. Emerging studies have highlighted the potential protective effects of the Mediterranean diet against stroke, yet the magnitude of this association and the underlying mechanisms remain to be fully clarified. Some epidemiological studies have demonstrated a significant inverse relationship between adherence to the Mediterranean diet and stroke risk, particularly ischemic stroke [8–15]. However, variations in the diet's effectiveness based on sex, age, and population subgroups have been observed, suggesting the need for a more nuanced understanding of its protective effects [8–15]. There is evidence suggesting that the protective effects of the Mediterranean diet may vary by sex, potentially due to differences in hormonal regulation and cardiovascular physiology. For instance, women may experience greater benefits from the diet’s anti-inflammatory properties during postmenopause, a period associated with increased vascular inflammation. These findings underscore the need for sex-specific analyses in future research to tailor dietary interventions more effectively. One critical area that warrants further exploration is the variation in the composition of the “Mediterranean diet” across different countries. The traditional Mediterranean diet is not a monolithic entity but rather a collection of dietary habits that vary significantly across the Mediterranean region [30–35]. These regional differences could contribute to the heterogeneity observed in studies assessing the diet’s impact on stroke risk. Moreover, the confounding effects of other aspects of the Mediterranean lifestyle, such as physical activity levels, social interactions, and overall lifestyle habits, further complicate the ability to draw definitive conclusions. These lifestyle factors, integral to the Mediterranean way of life, may synergistically enhance the diet’s protective effects, making it challenging to isolate the diet's specific impact on stroke prevention.

Given these complexities, the goal of this study was to conduct a comprehensive meta-analysis that synthesizes data from a diverse range of studies to clarify the relationship between adherence to the Mediterranean diet and stroke risk. By systematically analyzing these studies, this meta-analysis aims to provide robust evidence on the effectiveness of the Mediterranean diet in stroke prevention, accounting for potential sources of heterogeneity and confounding factors.

## Methods

### Study selection

A comprehensive search for relevant studies was conducted in the PubMed, Web of Science, and Google Scholar databases from 1990 until 2024. The search utilized keywords combined as detailed in Table 1: “Mediterranean diet” or “dietary patterns,” in combination with “stroke,” “cerebrovascular disease,” or “ischemic—or hemorrhagic stroke.” The literature search was limited to full-text publications without language restrictions. Additionally, the reference lists of identified articles and related previous meta-analyses were reviewed to uncover any further studies.

**Table 1:** List of keyword combinations for research on the relationship between Mediterranean diet adherence and stroke risk

Combination	Keywords
1	“Mediterranean Diet” AND “Stroke”
2	“Mediterranean Diet” AND “Cerebrovascular Disease”
3	“Mediterranean Diet” AND “Ischemic Stroke”
4	“Mediterranean Diet” AND “Hemorrhagic Stroke”
5	“Dietary Patterns” AND “Stroke”
6	“Dietary Patterns” AND “Cerebrovascular Disease”
7	“Dietary Patterns” AND “Ischemic Stroke”
8	“Dietary Patterns” AND “Hemorrhagic Stroke”

### Eligibility criteria

**Fig. 1:**
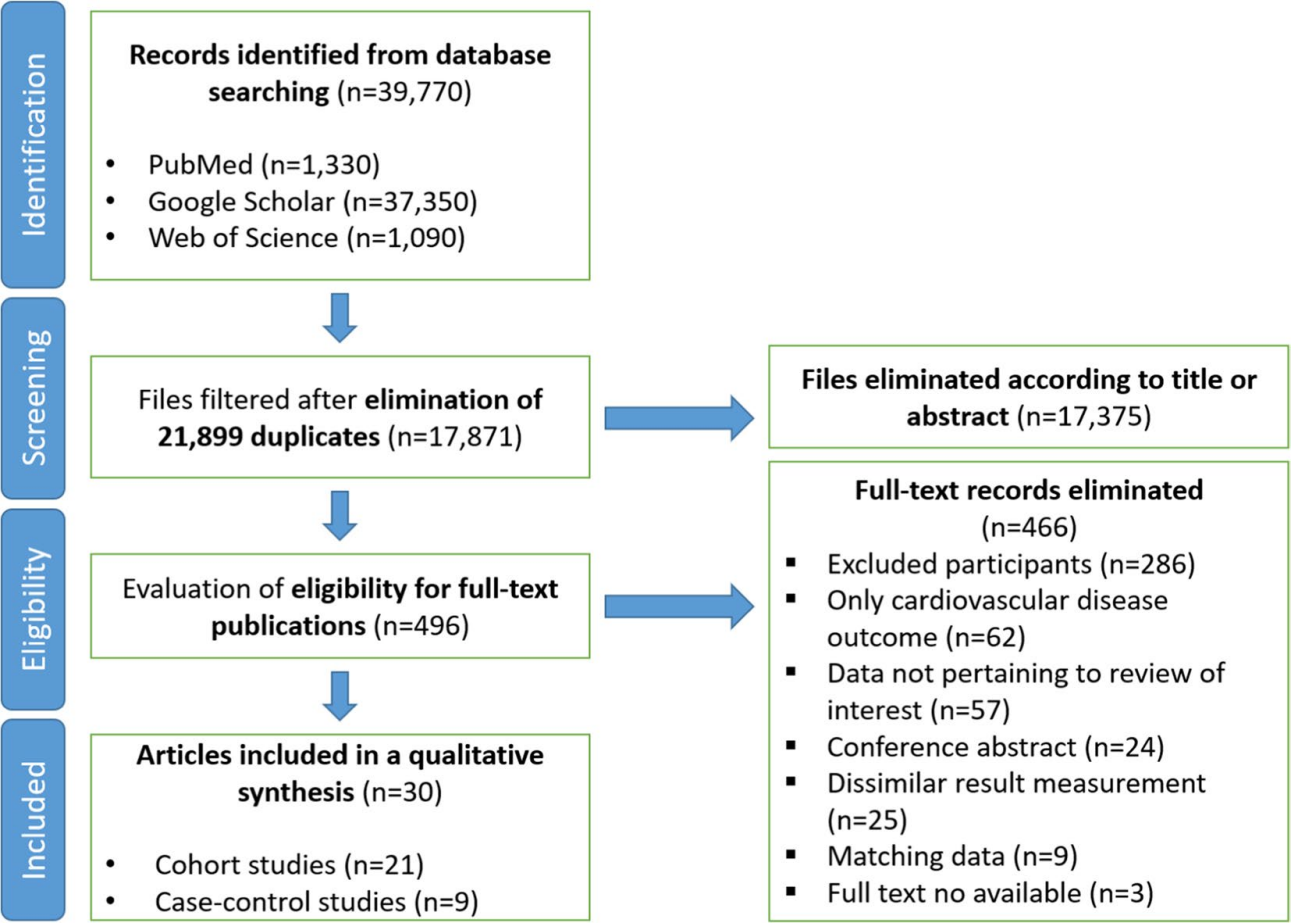
Flow diagram illustrating the article selection process

Two independent reviewers (AL, MF) screened all records and included studies that met the criteria listed in Table 2. In addition, Fig. 1 presents the study selection process for the meta-analysis.

**Table 2:** Eligibility criteria for study selection

Criterion	Description
Study population	Adults adhering to the Mediterranean diet (with comparisons to those following other dietary patterns or general populations)
Exposure of interest	Adherence to the Mediterranean diet, assessed through dietary questionnaires or other validated measures
Reported estimates	Studies reporting relative risk (RR), hazard ratios, or odds ratios and 95% confidence intervals (CIs) for stroke risk, or where these could be calculated from provided data
Outcome	Incidence of stroke, including subtypes such as ischemic stroke and hemorrhagic stroke
Study design	Observational studies, including cohort or case–control designs. In cases of duplicate studies from the same population, the study with the longest follow-up period was included

### Evaluating the overall effect

We conducted our statistical analysis using an updated approach available through the web platform at https://metaanalysisonline.com. To estimate pooled hazard ratios (HR), odds ratios (OR), and their 95% confidence intervals (CI), we employed a random-effects model. We used forest plots to visualize the results of individual studies and their aggregate effect, offering a comprehensive view of data variability and the overall effect size. To assess heterogeneity among the included studies, we applied the chi-squared test and calculated the *I*^2^ statistic.

### Analysis of publication bias

Funnel plots were constructed to evaluate the relationship between study effect estimates and their precision, which also allowed us to examine the potential presence of publication bias. Egger’s regression test was performed to statistically assess the significance of any observed bias.

### Assessing sample size adequacy

To determine the adequacy of the sample size, we performed a trial sequential analysis (TSA). The a priori required information size (APIS) was calculated assuming a 10% relative risk reduction, with a two-sided α of 5% and a statistical power (1 – β) of 80%. TSA was executed using Stata 17.0 with the *metacoumbounds* package. A Z-curve plot was generated to depict the relationship between actual cumulative sample size (AIS), time, and cumulative Z-scores. This analysis was crucial in determining whether the existing sample size was adequate to draw conclusive inferences or if further studies would be needed to solidify the findings.

## Results

### Identification of studies

In the initial phase of our systematic review, a total of 39,770 records were identified across multiple databases, including PubMed (*n* = 1330), Google Scholar (*n* = 37,350), and Web of Science (*n* = 1090). After removing 21,899 duplicates, 17,871 unique records were subjected to a title and abstract screening. This process led to the exclusion of 17,375 records that did not meet our inclusion criteria. Subsequently, the full texts of 496 articles were assessed for eligibility. During this stage, 466 articles were excluded for various reasons, such as studies only focused on cardiovascular disease (*n* = 62), or other methodological issues like lack of pertinent data (*n* = 57) or being a conference abstract only (*n* = 24). Ultimately, 30 articles were included in the qualitative synthesis, comprising 21 cohort studies and nine case–control studies (see Fig. 1).

### Cohort studies

All together 21 studies were analyzed [36–56]. Based on the analysis performed using random-effects model with inverse variance method to compare the hazard rate (HR), there is a statistical difference, the summarized hazard rate is 0.88 with a 95% confidence interval of 0.84—0.91. The test for overall effect shows a significance at *p* < 0.05.

**Fig. 2:**
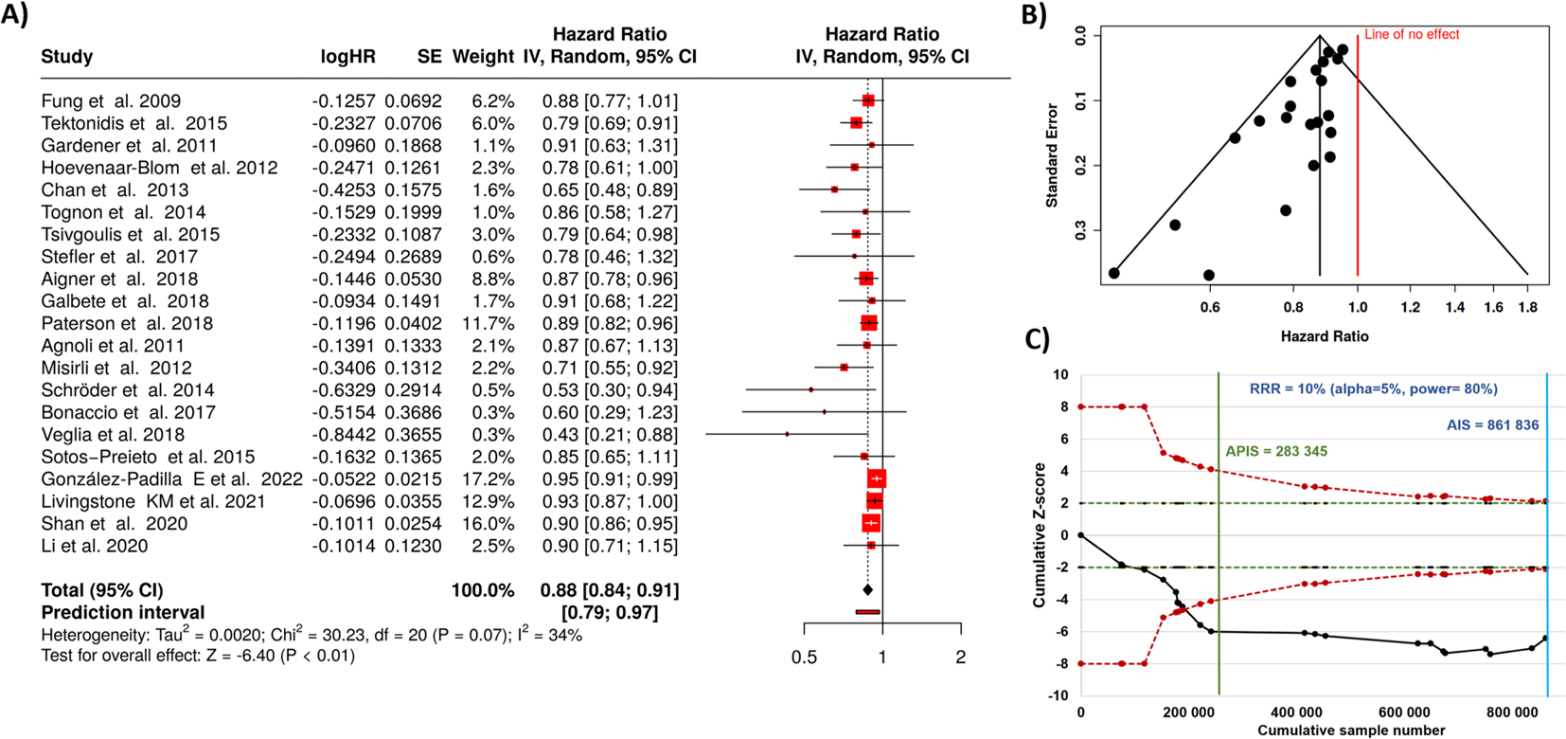
Results for all cohort studies comparing Mediterranean diet and stroke risk. There is a highly significant reduction in stroke incidence with a total HR of 0.88 (**A**). On the other hand, the funnel plot indicates a potential publication bias (**B**). The Z-score plot of cohort studies investigating the correlation indicates that no additional studies are needed to get a definitive conclusion (**C**). SE, standard error; CI, confidence interval; IV, inverse variance; APIS, a priori information size; AIS, actual information size; RRR, relative risk ratio

Notably, a noteworthy heterogeneity was detected (*p* = 0.07), suggesting inconsistent effects in magnitude and/or direction. The *I*^2^ value indicates that 34% of the variability among studies arises from heterogeneity rather than random chance (as shown in Fig. 2A).

The funnel plot indicates a potential publication bias. Egger’s test supports the presence of funnel plot asymmetry (intercept: − 1.35, 95% CI: − 1.86 to − 0.84, t: − 5.209, *p*-value: 0, see Fig. 2B).

The trial sequential analysis, as depicted in the Z-score plot in Fig. 2C, demonstrated that the total cumulative sample size (*n* = 861,836) was higher than the sample number necessary to draw definitive conclusions (*n* = 283,345).

### Case–control analysis

When combining all case–control studies, we were able to analyze all together nine trials [57–65]. Based on the analysis using random-effects model with the inverse variance method to compare the hazard rate, we observed a robust statistical difference, the summarized hazard rate was 0.54 with a 95% confidence interval of 0.4–0.73. In addition, the test for overall effect also supports significance at *p* < 0.05.

**Fig. 3:**
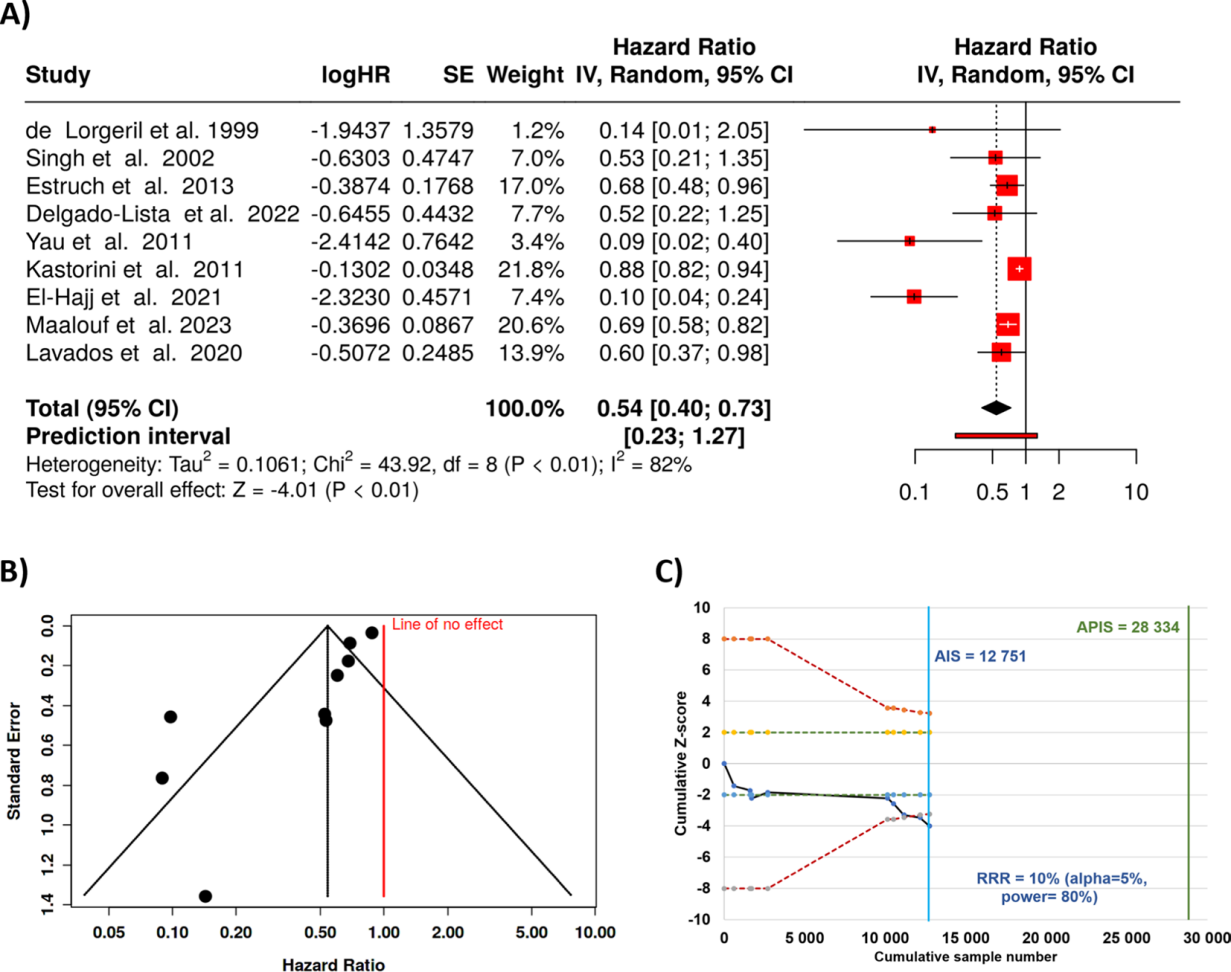
Case–control studies comparing Mediterranean diet and the risk of stroke. The forest plots show a highly marked effect with a HR of 0.54 (**A**). The funnel plot indicates a potential publication bias (**B**). Z-score plot of case–control studies investigating the correlation between obesity and colorectal cancer indicates the necessity of further studies to establish a definitive conclusion (**C**). SE, standard error; CI, confidence interval; IV, inverse variance; APIS, a priori information size; AIS, actual information size; RRR, relative risk ratio

On the other hand, a significant heterogeneity was detected (< 0.01), suggesting varying effects in scale and/or direction. The *I*^2^ value indicates that 82% of the unpredictability among trials stems from heterogeneity relatively to random chance (see Fig. 3A).

Similar to the incidence studies, the funnel plot points to a likely publication bias. The performed Egger’s test supports the existence of funnel plot unevenness (intercept: − 2.35, 95% CI: − 3.46 to − 1.24, t: − 4.135, *p*-value: 0.004, Fig. 3B).

The total number of cases included in the case–control analysis (*n* = 12,751) did not reach the a priori information size required for statistical significance (*n* = 28,334). This indicates that in this setting the current sample size is insufficient to draw definitive conclusions, necessitating further research to validate these findings. The Z-score plot utilizing all case–control studies which investigated the correlation between Mediterranean diet is provided in Fig. 3C.

## Discussion

This meta-analysis provides comprehensive evidence that adherence to the Mediterranean diet is associated with a significant reduction in stroke risk. The pooled hazard ratio of 0.88 from cohort studies indicates that individuals who closely follow the Mediterranean diet have a 12% lower risk of stroke compared to those who do not. This finding aligns with existing literature that has consistently highlighted the cardiovascular benefits of the Mediterranean diet, particularly its role in reducing the risk of ischemic stroke.

One of the key strengths of this study is its large sample size, which includes data from over 860,000 participants in the cohort studies and 12,751 participants in the case–control studies. The trial sequential analysis confirmed that the cumulative sample size of the cohort studies was sufficient to draw definitive conclusions, thereby strengthening the validity of our findings. However, it is important to acknowledge the significant heterogeneity observed in both the cohort (*I*^2^ = 34%) and case–control studies (*I*^2^ = 82%), which suggests variability in the effects of the Mediterranean diet on stroke risk across different populations and study designs.

The observed heterogeneity could be attributed to several factors [66], including differences in the definition and composition of the “Mediterranean diet” across studies. The Mediterranean diet is not a standardized dietary pattern but rather a collection of dietary habits that vary significantly across the Mediterranean region and beyond [67]. While the Mediterranean diet is characterized by core components, regional variations—such as the emphasis on specific foods like olive oil in Greece versus fish in Southern Italy—may influence its overall impact. Many Mediterranean countries are moving away from the traditional Mediterranean dietary pattern, while Northern European and other countries globally are increasingly adopting a Mediterranean-like diet [68, 69]. Outside of the Mediterranean region, adherence to the Mediterranean diet often reflects adaptations to local food availability and cultural preferences. In non-Mediterranean countries, olive oil is frequently substituted with other unsaturated fats, such as canola oil, while nuts and seeds are emphasized due to their availability. Similarly, the consumption of fatty fish, a key component of the Mediterranean diet, is adapted based on regional seafood options. These substitutions may impact the overall efficacy of the diet, as the bioactive compounds and nutrient profiles of alternative foods may differ. These compositional variations further contribute to the heterogeneity observed in studies examining the diet’s protective effects against stroke. These regional differences could lead to variations in the observed protective effects against stroke. Additionally, the confounding effects of other lifestyle factors integral to the Mediterranean way of life, such as physical activity, social interactions, and overall lifestyle habits, may also contribute to the heterogeneity. These factors are known to synergistically enhance the diet’s protective effects, making it challenging to isolate the specific impact of diet alone on stroke prevention. The need for standardized definitions and adherence measures in future studies is emphasized. Furthermore, the presence of potential publication bias, as indicated by the funnel plots and Egger’s test, suggests that studies with positive results might be overrepresented in the literature, which could inflate the perceived benefits of the Mediterranean diet. This limitation highlights the importance of future research, particularly in underrepresented populations and contexts. The case–control studies included in this meta-analysis demonstrated a pronounced effect (HR = 0.54), suggesting that the Mediterranean diet could be particularly beneficial in specific populations or under certain conditions. However, while the cohort studies demonstrated sufficient sample sizes, the case–control studies did not meet the a priori information size requirement, indicating the need for larger, well-designed studies to confirm these findings. The significant heterogeneity and the fact that the total sample size did not reach the a priori information size required for statistical significance indicate that these findings should be interpreted with caution. Further research with larger and more diverse cohorts is needed to confirm these results and to better understand the conditions under which the Mediterranean diet is most effective in reducing stroke risk.

The molecular and cellular mechanisms by which the Mediterranean diet exerts its vasoprotective effects, thereby reducing the risk of stroke, are likely multifaceted and interconnected [17, 20, 34, 70–73]. These effects are mediated through anti-atherogenic, antioxidative, anti-inflammatory, and anti-aging pathways, providing valuable insights into how the Mediterranean diet promotes healthy brain aging. Atherosclerosis, a chronic condition characterized by plaque buildup in arterial walls, is a key cause of ischemic stroke. The Mediterranean diet exerts potent anti-atherogenic effects, in part, by improving lipid metabolism [72]. Rich in monounsaturated fats from olive oil, the diet has been shown to reduce low-density lipoprotein (LDL) cholesterol while increasing high-density lipoprotein (HDL) cholesterol [72, 74–80]. Elevated LDL cholesterol is a major risk factor for atherosclerosis as it promotes plaque formation, whereas HDL cholesterol is protective. By improving lipid profiles, the Mediterranean diet reduces the likelihood of plaque formation and progression within arteries [81–85]. Additionally, the diet is high in omega-3 polyunsaturated fatty acids (PUFAs) from fish and nuts, which lower triglycerides and stabilize atherosclerotic plaques [86]. Omega-3 PUFAs inhibit pro-inflammatory gene expression and reduce inflammation in arterial walls, helping prevent plaque rupture, which is critical in stroke prevention [87]. Furthermore, the diet’s antioxidant-rich foods, including fruits, vegetables, and olive oil, help prevent LDL oxidation, a key event in atherosclerosis [75, 77, 88]. Oxidized LDL is more readily taken up by macrophages, forming foam cells that accelerate plaque development. By reducing oxidative LDL damage, the Mediterranean diet slows atherogenesis.

Aging-induced microvascular impairment is a critical factor in the pathogenesis of ischemic brain damage [89]. As individuals age, there is a gradual reduction in microvascular density, known as microvascular rarefaction, which compromises the ability of small blood vessels to supply oxygen and nutrients to brain tissue. This can be exacerbated by other cardiovascular risk factors, such as obesity, hypertension, and hyperhomocysteinemia. This reduction in microvascular density, coupled with aging-related endothelial dysfunction and impaired cerebral blood flow (CBF) regulation, exacerbates the brain’s vulnerability during ischemic events. Endothelial dysfunction impairs vasodilation and promotes vasoconstriction, which further limits blood flow to areas at risk during stroke [89]. Additionally, the brain’s ability to regulate CBF in response to metabolic demands becomes compromised with age [90], making it more difficult to preserve tissue in the peri-infarct region during ischemia [91]. The Mediterranean diet may play a significant role in mitigating these effects and improving microvascular function in the brain. By protecting against microvascular impairment, the Mediterranean diet may help lessen the extent of ischemic damage during stroke, ultimately reducing stroke severity and improving outcomes.

Hypertension is another major stroke risk factor, and the Mediterranean diet aids in regulating blood pressure [92–95]. The diet promotes potassium intake while reducing sodium consumption, essential for maintaining healthy blood pressure. In addition, the Mediterranean diet enhances NO bioavailability, promoting endothelium-mediated vasodilation and lowering blood pressure [96–100]. Better endothelial function not only aids in blood pressure regulation but also reduces the risk of clot formation, further lowering stroke risk [101]. The Mediterranean diet’s emphasis on whole foods, healthy fats, and nutrient balance supports weight management [102], which is also important for blood pressure control and stroke prevention, as obesity-related metabolic syndrome is a known risk factor for both.

The Mediterranean diet’s anti-inflammatory properties are another key factor in stroke prevention [103, 104]. Chronic inflammation drives the progression of atherosclerosis and other vascular diseases, with inflammatory markers such as C-reactive protein (CRP) and interleukin-6 (IL-6) linked to plaque instability and rupture [105–107]. The Mediterranean diet’s rich content of omega-3 fatty acids, polyphenols, and fiber lowers systemic inflammation, reducing these markers and mitigating the inflammatory processes that contribute to vascular disease [103, 104]. Polyphenols, including resveratrol, activate endogenous antioxidant defenses, such as Nrf2-regulated antioxidative responses and SIRT1-mediated cellular stress resilience pathways [108, 109], and reduce both mitochondria-derived and NADPH oxidase derived ROS production, attenuating cellular oxidative stress and inhibiting redox sensitive pro-inflammatory signaling mechanism. Omega-3 fatty acids shift immune cells toward an anti-inflammatory state, reducing their contribution to atherosclerosis [110, 111]. Additionally, the diet’s fiber content promotes gut health, fostering the production of short-chain fatty acids [112–114], which have systemic anti-inflammatory effects.

The Mediterranean diet also exerts anti-aging effects, further enhancing its role in reducing stroke risk [18, 115]. Aging contributes to the development of atherosclerosis [116] and stroke through mechanisms such as oxidative stress, heightened state of inflammation [105–107], mitochondrial dysfunction [117–120], and cellular senescence [121]. The Mediterranean diet reduces oxidative stress, a key factor in vascular aging, by providing antioxidants that neutralize ROS [17, 122–124]. This protects the endothelium from oxidative macromolecular damage and its downstream consequences as well from activation of redox sensitive pro-inflammatory, pro-atherogenic pathways. Oxidative stress impairs endothelial function by depleting NO, leading to vasoconstriction and a higher risk of thrombosis. By preserving NO availability and reducing ROS levels, the Mediterranean diet promotes endothelial health contributing to protection against stroke.

Mitochondrial dysfunction, characterized by dysregulated expression of the mitochondrial electron transport chain, reduced energy production, increased ROS generation and decreased mitochondrial antioxidant defenses, accelerates aging and contributes to both macrovascular diseases [125, 126] and microvascular pathologies [127] and endothelial functional impairment [128]. The Mediterranean diet enhances mitochondrial efficiency and promotes biogenesis through components like polyphenols [18, 115]. Omega-3 fatty acids also help maintain mitochondrial function and membrane integrity [115, 129], reducing oxidative damage associated with aging. Additionally, the anti-inflammatory and antioxidant properties of the Mediterranean diet likely help reduce the accumulation of senescent cells [18, 122], which secrete pro-inflammatory factors contributing to the pathogenesis of atherosclerosis [116, 130–134]. Autophagy, a process by which cells remove damaged components and recycle them for energy, plays a key role in aging and vascular health [135–138]. Components of the Mediterranean diet have been shown to enhance autophagy [139–141], promoting cellular repair and reducing the buildup of damaged proteins and organelles that contribute to cellular aging. In conclusion, the Mediterranean diet exerts its vasoprotective effects through a combination of anti-atherogenic, antioxidative, anti-inflammatory, and anti-aging mechanisms. These interconnected processes—improved lipid metabolism, blood pressure regulation, enhanced endothelial function, reduced inflammation, and support of mitochondrial health—contribute to the diet’s ability to prevent atherosclerosis and reduce the risk of stroke.

The Mediterranean diet’s protective effects may also be mediated through epigenetic mechanisms [18, 142–144]. Key components of the diet have been shown to influence gene expression by modulating epigenetic markers, including DNA methylation and histone acetylation [142]. These modifications play a crucial role in regulating pathways associated with inflammation, oxidative stress, and vascular function. For example, polyphenols like resveratrol can activate SIRT1, a histone deacetylase linked to improved endothelial function and reduced oxidative damage [145, 146]. By targeting fundamental pathways involved in epigenetic regulation of cellular aging processes, the Mediterranean diet provides an additional layer of protection against stroke and other age-related diseases [143, 144].

The protective effects of the Mediterranean diet, as demonstrated by the findings of this meta-analysis, have substantial public health implications. The consistent evidence supporting the Mediterranean diet’s role in reducing stroke risk highlights its potential as a powerful preventive strategy. Given the significant burden of stroke, particularly in countries like Hungary [1–3], where stroke mortality rates are among the highest in Europe, promoting adherence to the Mediterranean diet could become a pivotal element in public health initiatives aimed at reducing stroke incidence and mortality. According to the World Health Organization, stroke is the third leading cause of death in the country, with an age-standardized mortality rate that underscores the severity of the problem [1–3]. The high prevalence of stroke in Hungary reflects broader issues related to unhealthy aging, particularly poor dietary habits, which are key contributors to the country’s cardiovascular disease burden [147–152]. In response to this crisis, the Semmelweis Study—a prospective workplace cohort study—was initiated to investigate the underlying causes of unhealthy aging and to develop targeted interventions [153]. One such intervention is the Semmelweis-EUniWell Workplace Health Promotion Program, which seeks to address the specific causes of unhealthy aging, with a focus on improving dietary habits. By clarifying the relationship between the Mediterranean diet and stroke risk, this meta-analysis offers critical insights that will inform the development and refinement of both the Semmelweis Study and the Workplace Health Promotion Program. Integrating the Mediterranean diet into such national programs has the potential to address dietary inadequacies, particularly in the workplace setting, where interventions can reach large segments of the population.

In conclusion, this meta-analysis presents compelling evidence that adherence to the Mediterranean diet is associated with a substantial reduction in stroke risk. While the results are encouraging, further research is necessary to address the observed heterogeneity and investigate the mechanisms underlying the diet’s protective effects. Continued exploration in this area will be crucial for developing tailored dietary recommendations and public health strategies to mitigate the global burden of stroke.
